# The Antioxidant Role of a Taurine-Enriched Diet in Combating the Immunotoxic and Inflammatory Effects of Pyrethroids and/or Carbamates in *Oreochromis niloticus*

**DOI:** 10.3390/ani11051318

**Published:** 2021-05-05

**Authors:** Amany Abdel-Rahman Mohamed, Afaf N. Abdel Rahman, Gamal A. Salem, Maha M.El Deib, Mohamed A. Nassan, Nasreddin R. Rhouma, Safaa I. Khater

**Affiliations:** 1 Department of Forensic Medicine and Toxicology, Zagazig University, Zagazig 4511, Egypt; 2 Department of Fish Diseases and Management, Faculty of Veterinary Medicine, Zagazig University, Zagazig 44511, Egypt; 3 Department of pharmacology, Faculty of Veterinary Medicine, Zagazig University, Zagazig 44511, Egypt; gamal_vet_85@yahoo.com; 4 Department of Drug Technology, Faculty of Medical Technology, Al-Jufra University, Houn 61602, Libya; 5 Department of Biochemistry, Faculty of Veterinary Medicine, Zagazig University, Zagazig 4511, Egypt; dr.mmeldeib@yahoo.com (M.M.E.D.); safaa_khater83@yahoo.com (S.I.K.); 6 Department of clinical laboratory sciences, Turabah University College, Taif University, P.O. Box 11099, Taif 21944, Saudi Arabia; m.nassan@tu.edu.sa; 7 Department of Biology, Faculty of Science, Misurata University, Misurata 2478, Libya; nasser_micro@yahoo.com

**Keywords:** lambda-cyhalothrin, methomyle, taurine, *Oreochromis niloticus*, immunity, growth, CXC, IL-1β, TNF-α

## Abstract

**Simple Summary:**

Insecticidal pollution of surface waters is known to hurt the growth, survival, and breeding of aquatic animals. Different types of insecticides are known to be toxic to different aquatic organisms, particularly to fish species. In different types of wastewater, the fishes get exposed to different mixtures of insecticides. The current study hypothesized that co-exposure to lambda-cyhalothrin (LCT) and methomyl (MTM) insecticides might be more harmful due to duplicated effects than exposure to either one of them at a time. *Oreochromis niloticus* was the target fish in this study. The combative roles of taurine (TUR) against LCT and MTM exposures were evaluated. In the present work, exposure of *O. niloticus* to LCT and/or MTM exhibited adverse effects on immunological parameters, including leukocyte count, complement 3 concentration, antioxidant enzyme concentrations, and mRNA expression for cytokines (TNF-α and IL-1β) and chemokines (CC and CXC). This study also elucidated the more severe toxic effect of LCT than exposure to MTM in *O. niloticus* fish. The immune response and growth performance of *O. niloticus* showed marked improvements when provided a 1% TUR-enriched supplement.

**Abstract:**

Indiscriminate use of insecticides is a major concern due to its ubiquitous occurrence and potential toxicity to aquatic animals. This study investigated the adverse effects of lambda-cyhalothrin (LCT; C_23_H_19_ClF_3_NO_3_) and methomyl (MTM; C_5_H_10_N_2_O_2_S) on immune system modulations and growth performance of juvenile fishes. The supportive role of a taurine (TUR; C_2_H_7_NO_3_S)-supplemented diet was also evaluated. Juvenile *O. niloticus* fishes were exposed to LCT (0.079 µg/L), MTM (20.39 µg/L), or both in water and were fed on a basal diet only or taurine-supplemented basal diet. Exposure to LCT and MTM retarded growth and increased mortality rate. LCT and MTM reduced antioxidant enzyme activities (superoxide dismutase and glutathione peroxidase) and innate and humoral immunity but upregulated interleukin and chemokine expressions. Moreover, exposure to LCT and MTM elevated 8-OHdG levels and increased the mortality of *Oreochromis niloticus* after the experimental bacterial challenge. The TUR-enriched diet enhanced antioxidant enzymes and acted as a growth promoter and anti-inflammatory agent. TUR can modify innate and adaptive immune responses. Furthermore, TUR supplementation is a beneficial additive candidate for mitigating LCT and MTM toxicities mixed with *O. niloticus* aquafeed.

## 1. Introduction

The extensive use of pesticides in safeguarding our crops from pests has some noxious impacts on the aquatic environment, including on fish. Pesticides find their way into water bodies through runoffs, drifts, or leaching [1]. Fishes are mostly affected by these hazards through absorption of these pesticides through their skin and uptake by gills; the pesticides bioaccumulate in their tissues [2,3]. There are many types of pesticides, such as insecticides, herbicides, and fungicides. Insecticides can be grouped into various families, such as organophosphates (chlorpyrifos-ethyl, profenofos, malathion, etc.) [4], pyrethroids (cypermethrin (CP), lambda-cyhalothrin, etc.), and carbamates (methomyl, carbaryl, propoxur, carbofuran, etc.) [5]. Unfortunately, synthetic pyrethroids are sprayed and bioaccumulated in the aquatic environment by directly spraying water sources, and via farm runoff and forests, due to their high absorption. In contrast with birds and humans, they have an expanded toxic effect on various fish species and water invertebrates (1000 times). Lambda-cyhalothrin (LCT) is a new breed of type II pyrethroid insecticide. It is preferred over other insecticides owing to its superb potencies, greater photo-stability, and potential toxicity to a wide variety of insect pests [6,7]. LCT has very low water solubility and fat solubility, and is extensively used to control agricultural and domestic pests [8]. The introduction of LCT into an aquatic system can bio-concentrate and disrupt the biodiversity of organisms [9,10]. LCT was found to be highly concentrated (346 ng/L) in the surface water of Greek rivers and reached up to 797 ng/L concentration levels in agricultural regions of southern United States [11,12]. It was found to have similarly high concentrations in the tropical areas of the world, including soybean regions in Argentina, Brazil, and Paraguay (6.09, 5, and 16.57 µg/kg, respectively [13,14]. Lambda-cyhalothrin is very toxic to fish [8]. Sublethal concentrations of LCT can cause oxidative stress and DNA damage [15]. Furthermore, in *Channa punctatus* and *Clarias batrachus,* LCT caused modulation of the level of amino acid catabolism and rescued nitrogen metabolism during oxidative deamination and transamination [16], and caused a significant decline in *Clarias batrachus* in stress biomarker enzyme activities [17]. Exposure to concentrations ranged from 0.005 to 0.5 µg/L, which are considered insignificant compared with those found even in surface water or sediment; several studies reported genetic damage, neurological impairments, oxidative injury, and osmoregulatory defects induced in different organs and tissues of *Prochilodus lineatus* fish [18]. LCT was also known to cause behavioral changes, a change to the color of the skin, hyperactivity, enhanced balance loss, fast swimming, more intensified underwater movement, opercular and surface speed, and seizures [19].

Methomyl is one of the carbamate insecticides used worldwide [20]. Methomyl (MTM), *S*-methyl-1-*N*-[(methyl carbamoyl)-oxy]-thioacetimidate, is popularly used in agricultural countries because of its high efficiency, strong solubility, and broad biological activity [20]. MTM is used against many insects through systemic poisoning. It works through direct contact on insects, mites, ticks, and spiders [21]. High levels of MTM have been detected in water bodies and foods [22]. MTM exposures pose a significant health risk and result in toxicity for various aquatic species, including common carp [23,24]. MTM—which is essential in numerous vertebrate biomass ecosystems and substantial as a bioindicator for agrochemical pollution by, for example, insecticides—is extremely toxic to non-target organisms, including amphibians [25]. This insecticide is highly soluble in water and has low to moderate solubility in soil [26]. Carbamates and pyrethroids are the third and fourth largest major groups of insecticides that could be effectively used as alternatives for organophosphorus insecticides in agriculture.

A fish is a perfect model for studying the toxicity of pesticides in aquatic systems on account of the high sensitivity of fish to pesticides, their ability to metabolize xenobiotics, and their bioaccumulation rate [27]. Globally, *Oreochromis niloticus* L. is an essential and popular freshwater fish species among farmers because of its rapid growth, high nutritional value, palatability, and high market value [28]. Several severe forms of damage in many biological and biochemical processes of fish organs are induced by pesticide exposure, resulting in behavioral disorders, growth and reproductive retardations, and histological alterations in many vital organs [29]. LCT is highly toxic to the *O. niloticus* fingerlings, causing behavioral, neural, immunological, and oxidative impacts [5,30]. Moreover, a previous study on MTM insecticides revealed their genotoxic effects in *O. niloticus* [31].

Natural products’ evolution as supplements for maintaining fish health has gained significant attention [27,29]. Taurine (TUR) is a sulfonic amino acid that accounts for 30–50% of the free amino acid pool in animal tissues [32]. It is present in considerable amounts in fish meals and is absent in plant-based proteins [33]. Many fishes cannot synthesize TUR and depend primarily on dietary supplementation [34]. TUR is concerned with many physiological functions, such as modulation of immunity, detoxification, anti-oxidation, muscular and neural development, and endocrine functions [35,36]. In fishes, the synthesis and benefits of TUR depend on fish species, size, and feeding habits [37]. TUR was reported to enhance the growth performance and feed utilization of different fish species [38,39,40].

Due to the previously-mentioned evidence regarding the dangerous effects of LCT and MTM, they present a massive risk because of their availability in aquatic ecosystems, even in surface water or as residues in fish tissues, which could be transferred to humans or animals and aquatic organisms even through direct consumption or via fish meals. There is very scarce published literature on the single or combined exposure endpoints of LCT and/or MTM regarding *O. niloticus*. To achieve this objective, several immunological endpoints, including differential leukocyte count, complement 3 concentration, oxidative stress, and immune-related gene expression (TNF-α, IL-1B, and some chemokines (CC and CXC)) were estimated. Additionally, resistance to *Aeromonas hydrophila* was studied. The research also attempted to assess the residual concentrations of these insecticides in organs and tissues of fish to create a key tool for risk assessments of them; furthermore, the economic view was monitored by detecting the growth performance of fish at the end of the experimental period. The combative role of dietary supplements is one of the prominent roles of scientific research, so the possible supportive role of TUR dietary supplementation on the threats of LCT, MTM, or both of *O. niloticus* was studied, for the first time to our knowledge, based on the unique beneficial biological properties of TUR and its availability as a supplement in aquafeeds that contain high levels of plant protein sources only. Accordingly, this study aimed to investigate the ability of TUR to perform different roles through enhancing growth, combating immunosuppression, enhancing immune encoding gene expression patterns in the spleen, increasing oxidative damage, and increasing the inflammatory response against LCT and/or MTM.

## 2. Materials and Methods

### 2.1. Chemicals

Lambda cyhalothrin technical grade (98% purity) [(*R*)-cyano-(3-phenoxyphenyl) mthyl](1*S*,3*S*)-3-[(*Z*)-2-chloro-3,3,3-trifluoroprop-1-enyl]-2,2-dimethylcyclopropane-1-carboxylate was purchased from Sigma Aldrich (St. Louis, MO, USA). Methomyle (methyl *N*-(methylcarbamoyloxy) ethanimidothioate (97%) was purchased from Sigma Aldrich (St. Louis, MO, USA).

### 2.2. Fish

The Fish Hatchery of the Central Laboratory for Aquaculture Research in Abbassa, Egypt, supplied *O. niloticus* weighing 30.12 ± 0.003 g. Polyethylene bags containing one-third (about 10 L) with dechlorinated water and two-thirds air-enriched water (2/3) were used to carry the fishes to the laboratory. During the experiment, glass aquaria containing 75 L of dechlorinated tap water were used to house the fishes. In every aquarium (80 × 40 × 30 cm), approximately 30% of the water was siphoned about three times a week to remove excreta by completely submerging the tube inside the aquarium so that it filled with water and dropped the amount to be replaced out of the aquarium. This could be done easily by keeping the tube at a diagonal angle with the tube opening pointed upwards and equipped with a central air compressor-linked air stone to maintain continuous ventilation for each tank. The fishes were exposed to a 12 h/12 h (light and obscurity) light-dark period. The average parameters of water quality for dissolved oxygen were 6.97 ± 0.06 mg L^−1^ and temperature 26.20 °C ± 0.12 °C; pH was 6.66 ± 0.02 mg L^−1^; nitrite 15 ± 0.03; ammonia 0.13 ± 0.01; nitrate 2.45 ± 0.06 mg L^−1^. The basal diet ingredient preparation and its chemical analysis were (Table 1) conducted according to National Research Council [41]. The ingredients were mechanically blended and pelletized using a meat mincer that was equipped with a 1.5 mm die diameter. The pellets were air-dried and stored at 4 °C in a refrigerator until further use. After 14 d of acclimatization, the experiment was supervised by the Animal Use in Research Committee, and performed at the Fish Diseases and Management Department Laboratory, Faculty of Veterinary Medicine, Zagazig University, Egypt. In scientific investigations with ZU-IACUC/2/F/68/2020, all experimental procedures were conducted in compliance with the ethical guidelines approved by the National Institutes of Health for Use and Treatment of Laboratory Animals.

### 2.3. Experimental Design

#### 2.3.1. Experiment 1 (Acute Toxicity Study)

The acute toxicity study was performed to detect exactly the 96-h LC_50_ of LCT and MTM in *O. niloticus*. Each compound’s stock solution was prepared in water at the concentration of 5.62 µg/L for LCT or 846.32 µg/L for MTM. The stock solution was used subsequently for the preparation of the different concentrations used in this study. A static 96-h acute toxicity test was conducted on 100 *O. niloticus* fishes for each compound: LCT and MTM. Seven groups (10 fish/group) were exposed to seven different concentrations (0.07, 0.92, 1.21, 1.45, 1.66, 1.96, and 2.18 µg/L) of LCT, and a control group (10 fish) was exposed to clean dechlorinated tap water for 96 h. At the same time, MTM concentrations were 160.0, 170.0, 210.0, 310.0, 380.0, 560.0, and 640.0 µg/L, respectively, in the group. The fish received no food, and water was not exchanged during the experiment. The number of mortalities was recorded at different time points: 24, 48, 72, and 96 h; the dead fish were immediately removed, and the clinical signs and postmortem lesions were recorded. Finney’s probit analysis [42] was used to calculate the 96-h LC_50_ value and 95% confidence interval limits using EPA, LC_50_ Software Program, version 1.50. The 96-h LC_50_ [43] value of LCT was determined as 1.59 µg/L and for MTM (407.94 µg/L) for *O. niloticus*.

#### 2.3.2. Experiment 2 (Subacute Toxicity Study)

Four hundred and eighty apparently healthy *O. niloticus* fingerlings were randomly assigned into eight groups (60 fish each group), each with three replicates (20 fish/replicate). The first group served as the control and was fed on a basal diet. The second group was fed on basal diets enriched with 1% TUR (10 g/kg). The third and fourth groups were fed on a basal diet and exposed to LCT 1/20 of LC_50_ (0.079 µg/L) and MTM 1/20 LC50 (20.39 µg/L), respectively, as shown in Table 2. The fifth group was exposed to a mixture of the two compounds (LCT + MTM). Meanwhile, the sixth (LCT + TUR), seventh (MTM + TUR), and eighth (LCT + MTM + TUR) groups were fed on a 1% TUR-enriched diet and at the same time exposed to LCT and/or MTM, respectively, at the aforementioned concentrations for 60 days. The water was wholly changed twice weekly, and freshly prepared pesticide solutions were added. The food was provided two times (9:00 a.m. and 6.00 p.m.) daily at a rate of 5% of their biomass. Thirty percent of the water from the bottom of the tank was siphoned daily to clear the excretory wastes.

### 2.4. Monitoring of Fish during the Experiment

The fish were monitored for any adverse clinical signs and mortalities and weighed every 14 days during the course of the experiment. To estimate the body weight gain (WG) in groups, body weight gain was evaluated with condition factor (K) and specific growth rate (SGR).

WG = Final mean bwt (g) − initial mean bwt (g);

K = (W × 100)/L^3^,

where W is the body weight (g), and L denotes length.

SGR = 100 [Ln final mean bwt in (g) − Ln initial mean bwt in (g)]/time intervals (days).

### 2.5. Blood and Tissue Sampling

Heparinized syringes were used to collect blood samples through puncture of the caudal vein for hematopoietic clinical indices, thereby obtaining approximately half a milliliter of blood from each fish and collecting it without anticoagulants to separate the serum. The serum was then used for measuring biomarkers by centrifugation in biochemical and immunological conditions at 3000 rpm/15 min. The fishes were sacrificed by spinal cord section. Liquid nitrogen quickly-frozen spleen and liver samples were collected and then preserved at −80 °C for extraction of total RNA and quantitative reverse transcription-polymerase chain reaction (RT-qPCR).

### 2.6. Evaluation of Hematological Indices

According to the Wintrobe process, the total number of different leukocytes was used manually. The Hema Screen 18 automatic hematology test (Hospitex Diagnostic, Sesto Fiorentino, Italy), RBC count (1op00/8 × 9p − 0/06/mL), mean cell volume (MCV), hematocrit value (PCV), and hemoglobin concentration (Hb) were calculated to be indicated as hematological indices [44].

### 2.7. Immunological Assay and Protein Profile

The commercially available ELISA kit was implemented according to the manufacturer’s instructions, and the serum concentrations of nitric oxide, immunoglobulin M (IgM), complement 3, and lysozyme activity were measured. For quantitative fractionation of serum proteins [4], the cellulose acetate electrophoresis (Helena Laboratories, Beaumont, TX, USA) approach was used.

### 2.8. Oxidative Stress Biomarkers in Serum and Acetyle Choline Esterase Enzyme (AchE) 

The superoxide dismutase (SOD) was estimated according to the method of Misra et al. [45], and glutathione peroxidase (Gpx) activity according to Paglia and Valentine [46]. Malondialdehyde (MDA) and the oxidative DNA damage were estimated according to the method used by Ohkawa*,* et al. [47]. The 8-hydroxy-2-deoxyguanosine (8-OHdG) marker level and acetylcholinesterase (AchE) activity were assessed using ELISA commercial kit (My-Biosource Inc., San Diego, CA, USA).

### 2.9. Transcriptional Profile Changes of Immune-Related Genes—Tumor Necrosis Factor α (TNF-α), Interleukin-1β (IL-1β), Interleukin-10 (IL-10), and CC and CXC Chemokines

RNeasy Mini Kit (Cat #74104, Qiagen, Germantown, MD, USA) obtained the total RNA from the spleen tissue at the end of the experimental time as per the manufacturer’s protocol. A Nano-Drop^®^ ND-1000 Spectrophotometer was used to determine the RNA extracted (NanoDrop Technologies, Wilmington, NC, USA). The first complementary DNA (cDNA) strand was synthesized with Revert-AidTM H Minus kits (Fermentas Life Science, Pittsburgh, PA, USA). A single microliter of synthesized cDNA was merged into a 12.5 µL SYBR^®^-Green PCR blend with Bio-Rad ROX, 5.5 µL of RNase-free water, and a reverse primer for each of the selected genes, 0.5 µL (10 pmol/µL). Unique primers were evaluated for optimum amplification at various concentration levels. Samples of cDNA were amplified in splenic tissues according to the unique primers—IL-1β, IL-10, TNF-α, CC-chemokine, and CXC-chemokine samples, as shown in Table 3 (Arisha et al., 2019). Initial denaturation was conducted at 95 °C for 15 min, accompanied by 40 cycles of 95 °C for 20 s, a mating at 60 °C for 30 s, and an extension at 72 °C for 30 s. Cycling conditions were used in PCR cycling. Target genes were normalized to glyceraldehyde-3-phosphate dehydrogenase (GAPDH) expression levels, and the relative fold changes in gene expression based on the 2−CT comparative method were measured [48].

### 2.10. Challenge Test

The known standard biochemical and pathogenicity profiles of *A. hydrophila* microbiological archives of the Department at Animal Health Research Institute, Dokki, Egypt, were collected. At 370 °C in tryptone soy broth, the bacteria were grown for 24 h. Then 800× *g* of the culture was centrifuged for 15 min at 40 °C. Suitable concentration was established in phosphate-buffered saline (PBS) at pH 7.2. The cells were washed with PBS. Any bacterial suspension was confirmed pathogenic using pathogenicity tests for the same species. The median lethal dose value (LD50) was estimated previously using Finney’s probit analysis method [42]. At the end of the experiment, after 60 days, a dose of 0.1 mL (1.5 to 1107 cells/mL) was randomly deposited in five fish/replicates (15 fish/group) and injected intraperitoneally [43]. After the injection, all irregular clinical signs and daily fish mortality were tracked daily over 15 days.

### 2.11. Data Analysis

Shapiro–Wilk normality (W) tests were performed to determine the normality and homogeneity of variances. One-way analysis of variance (ANOVA) was performed, and Tukey’s test was the post hoc test for comparing different experimental groups by GraphPad Prism version 8 (GraphPad Software Inc., San Diego, CA, USA). The data obtained are expressed as means ± SEs (standard error) in the current analysis. The statistically different groups were assessed when the *p*-value < 0.05.

## 3. Results

### 3.1. Growth Performance 

In terms of body weight gain, specific growth rate (SGR), and condition factor (K), substantial reductions (*p* < 0.001) in fish exposed to LCT were recorded in contrast to the control group, as shown in Table 4. The decreases in three growth efficiency parameters (weight gain, SGR, and K) in the co-exposed group were maximal. The addition of TUR to the fish diet by 1% noticeably enhanced weight gain. The feeding of fish with a TUR-supplemented diet simultaneously with the exposure to LCT and/or MTMs revealed different improvement degrees, as shown in Table 4. In comparing the control and TUR-groups, an increase in the weight gain was observed due to feeding on a TUR-enriched diet, which indicates the ability of TUR to enhance growth and mitigate the retarded growth caused by exposure to the insecticides implicated here in the current study (LCT and MTM).

### 3.2. Mortality Rate and Clinical Observations

**Figure 1:**
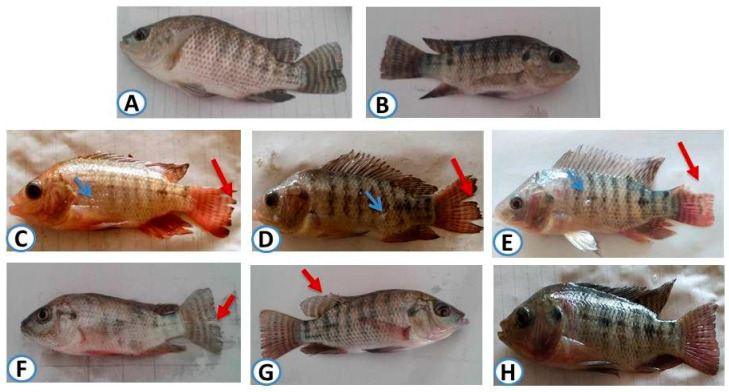
Clinical signs of *O. niloticus* that were exposed to LCT and/or MTM (1/20 of LC50) and fed on diets containing 1% taurine (TUR) for 60 days. (**A**,**B**) Fish that were fed on the basal diet, without or with TUR supplementation, showed normal appearance. (**C**–**E**) Fish that were fed on a basal diet only and exposed to LCT (**C**) or MTM (**D**) or both (**E**) showed severe fin rot (red arrow), increased mucus secretion (light blue arrow), erythema, and hemorrhages on different body parts. (**F**–**H**) Fish that were fed on a basal diet containing TUR and exposed to LCT (**F**) or MTM (**G**) or both (**H**) showed a normal appearance except slight fin rot (red arrow).

There were no reported mortalities in the control group or the TUR-supplemented groups during the experimental period (Table 4). On the contrary, the highest mortalities were recorded in the LCT + MTM-exposed group (61.67%) followed by LCT (51.67%) and MTM (43.33%)-exposed groups. Concurrent supplementation of *O. niloticus* with TUR protein with LCT and/or MTM decreased the mortality rates to 35, 30, and 20% in LCT + MTM, LCT, and MTM-exposed groups, respectively. The control and TUR groups exhibited no abnormal signs (Figure 1A,B). On the contrary, LCT and/or MTM-exposed fish showed loss of balance, erratic swimming, rapid opercular movement, dark body coloration, severe fin rot, increased mucus secretion, erythema, and hemorrhage on different body parts (Figure 1C–E). These signs showed significant improvements in groups of co-administered TUR with LCT and/or MTM (*p* < 0.001) (Figure 1F–H).

### 3.3. The Effects of Exposure to LCT and/or MTM and Their Combination on Hematological Variables

Hematological variables, including erythrogram and leukogram, were found affected in *O. niloticus* after exposure to LCT and/or MTM exposure for 60 days. There was a highly significant (*p* < 0.001) decrease in erythrograms (e.g., RBCs, packed cell volume (PCV), hemoglobin (Hb), and mean corpuscular volume (MCV)). Data showed that the exposure to both compounds caused the worst effect on hematological indices. However, the diet fortified with 10 g/Kg (1%) TUR could efficiently reverse the LCT and MTM-induced anemic index, but the improvement was the lowest after the combination exposure (LCT + MTM + TUR). Regarding leukograms, fish exposed to LCT and/or MTM showed high statistical significance (*p* < 0.001). Compared with values in the control group without any supplementation, total WBCs, heterophile, lymphocytes, eosinophils, and monocytes showed decreases. The total WBCs, eosinophils, heterophile, and lymphocytes were markedly reduced in the co-exposed group (LCT and MTM) compared with those of fishes exposed to LCT or MTM. The recovery was less when the combination of both LCT and MTM was given than when the TUR was concurrently given with each one of them separately (LCT + TUR) and (MTM +TUR), as shown in Table 4.

In this regard, the data presented in Table 5 indicate that the level of AchE in serum was significantly decreased (*p* < 0.001) after exposure to LCT and MTM for 60 days compared with control. Severe increases in the serum 8-OHdG due to the DNA oxidative damage occurred for LCT, MTM, and LCT + MTM.

### 3.4. Effects on AchE and 8-OHdG

MTM, LCT, and co-treated groups compared to the non-exposed groups (control and TUR) had elevated levels of 8-OHdG and reduced AchE enzyme activity. The groups received a TUR-supplemented diet concurrent with exposure to LCT and/or MTM, reduced the levels of 8-OHdG, and significantly (*p* < 0.001) activated the AchE activity.

### 3.5. Immunological Response Indices and Protein Profile 

The immunological response indices presented in Table 5 demonstrate that the innate and humoral (lysozyme, NO, and C3) regarding levels and activities in response to LCT exposure showed significant decreases. MTM alone or in combination with LCT revealed the exact effect of LCT as well. Additionally, TUR supplementation significantly (*p* < 0.001) elevated these parameters relative to LCT and/or MTM-exposed groups. Serum protein fractions in *O. niloticus* fish subjected to LCT and/or MTM and TUR-fortified diets for 60 days are tabulated in Table 5. Substantial declines in the total protein and globulin levels were observed as compared to the control group. The declines, including α-globulin, also occurred due to the pesticide exposure relative to the control group. In contrast to the control group, this decrease was attributed to a pronounced elevation of the albumin/globulin (A/G) ratio in the exposed groups, though no statistically significant (*p* < 0.001) difference was detected in albumin content between the experimental groups. TUR-supplemented diet minimized the decline in total protein and globulin levels as compared to the LCT and/or MTM values.

### 3.6. Properties of Oxidative Stress Indicators

**Figure 2:**
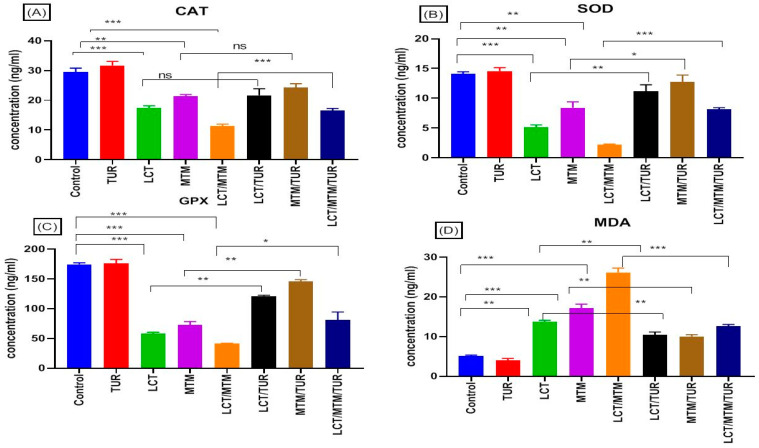
Changes in catalase (CAT), superoxide dismutase (SOD), and glutathione peroxidase (GPX), activities in the serum and the serum concentration of malonaldehyde (MDA) of *O. niloticus* after exposure to LCT and/or MTM (1/20 of LC50) and a diet containing 1% taurine (TUR) for 60 days. The values are shown as means ± SEs. One-way ANOVA was followed by Tukey’s post hoc tests to measure the significance of differences between groups. *—*p* ≤ 0.05 (low significance); **—*p* ≤ 0.01 (moderate significance); ***—*p* < 0.001 (highly significant difference). The comparisons are represented in the figure as follows: LCT versus LCT + TUR, MTM versus (MTM + TUR), and LCT + MTM versus LCT + MTM +TUR.

As illustrated in Figure 2, substantial decreases in antioxidant enzymes (catalase (CAT), superoxide dismutase (SOD), and glutathione peroxidase (GPx)) were evident even in fish exposed to or mixed with LCT and MTM, but a significant (*p* < 0.001) promotion of methyl diethanolamine (MDA), the lipid peroxidative damage product, was followed by a reduction in antioxidant enzymes. In the LCT group, the mean values were 17.4 ± 0.4, 5.1 ± 0.2, and 58.3 ± 1.02 ng/mL for CAT, SOD, and GPX, respectively, and 13.6 ± 0.2 for MDA, which was found elevated compared to the control group. In MTM group, the mean values were 21.3 ± 0.3, 8.4 ± 0.4, 72.7 ± 2.8, and 17.1 ± 0.5 ng/mL, respectively. In the co-exposed group (LCT+ MTM), the mean values were 11.25 ± 0.5, 2.2 ± 0.4, 41.1 ± 0.2, and 26.15 ± 0.7 ng/mL for CAT, SOD, GPX, and MDA, respectively. The values in the control group were 29.5 ± 0.6, 14.13 ± 0.5, 174.4 ± 1.3, and 5.1 ± 0.1 ng/mL, respectively. The supplementation with a TUR-enriched diet caused activation of the antioxidant enzymes, which were raised in the LCT + TUR group to be 21.6 ± 1.1, 11.2 ± 0.5, 121.3 ± 0.6, and 10.4 ± 0.3 ng/mL, respectively. The MTM + TUR group had 24.3 ± 0.6, 12.8 ± 0.5, 146.2 ± 0.6, and 10.03 ± 0.2 ng/mL levels of CAT, SOD, GPx, and MDA, respectively. The mean values in the LCT + MTM + TUR group for the four enzymes CAT, SOD, GPx, and MDA were 16.6 ± 0.3, 8.17 ± 0.1, 80.77 ± 0.6, and 12.6 ± 0.2 ng/mL, respectively.

### 3.7. Immune-Related Genes’ Relative Expression Changes

**Figure 3:**
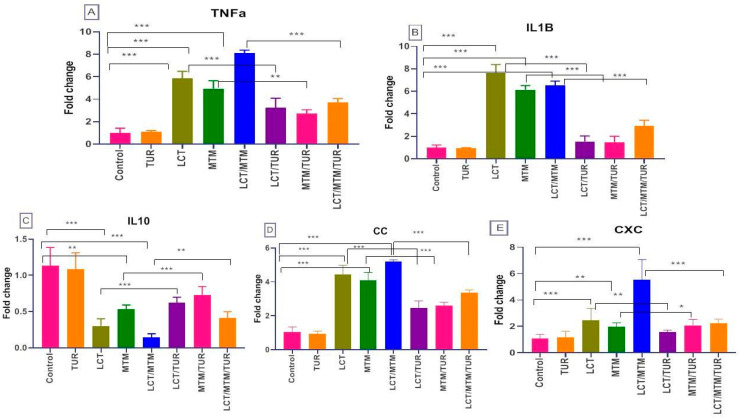
Fold changes in TNF-α, IL-10, IL-1β, CC, and CXC expression of *O. niloticus* after exposure to LCT and/or MTM (1/20 of LC50) and fed a diet containing 1% taurine (TUR) for 60 days. The values are shown as means ± SEs. One-way ANOVA followed by Tukey post hoc tests to measure the significance of differences between groups. *—*p* ≤ 0.05 (low significance); **—*p* ≤ 0.01 (moderate significance); ***—*p* < 0.001 (highly significant difference). The comparisons are represented in the figure as LCT versus LCT + TUR, MTM versus MTM + TUR, and LCT + MTM versus LCT + MTM +TUR.

The results of incorporating TUR in the diet, along with exposure to LCT alone or in combination with MTM of *O. niloticus* fish, in the expressions of TNF-α, IL-1β, CC, and CXC, are shown in Figure 3 for the splenic tissue. The expressions of these genes by qRT-PCR showed statistically significant (*p* < 0.001) down-regulation in the groups exposed to LCT by five-fold, but up-regulation of IL-10; and up-regulation for TNF-α—four-fold for IL-10, eight-fold for IL-1β, four-fold for CC, and two-fold for CXC. The mean values in the LCT group were 5.83 ± 0.37, 0.3 ± 0.06, 4.43 ± 0.32, 2.46 ± 0.52, and 7.63 ± 0.43 for TNFα, IL-10, CC, CXC, and IL-1β, respectively (control—1 ± 0.24, 1.13 ± 0.15, 1.08 ± 0.16, 1.1 ± 0.17 and 1 ± 0.13). The former increments were also found nearly in the same manner in the groups exposed to MTM alone (4.9 ± 0.40, 0.53 ± 0.03, 4.1 ± 0.26, 1.96 ± 0.18, and 6.13 ± 0.2186) or in combination with LCT (8.10 ± 0.15, 6.23 ± 0.04, 2.45 ± 0.24, 1.56 ± 0.09, and 1.53 ± 0.28, respectively). The up-regulation in the immune-related genes and chemokines reached the maximum standards in the co-exposed group regarding the control group. The feeding of fish with a 1% TUR-enriched meal restored the normal manner of expression in the combination groups, as shown in Figure 3, compared with the respective pesticide-exposed groups. In the LCT + TUR group, the values for TNFα, IL-10, CC, CXC, and IL-1β were 3.2 ± 0.41, 0.62 ± 0.04, 2.467 ± 0.24, 1.56 ± 0.09, and 1.53 ± 0.28, respectively. For LCT + MTM, they were 2.7 ± 0.21, 0.73 ± 0.06, 2.6 ± 0.11, 2.06 ± 0.27, and 1.47 ± 0.31. For LCT, MTM, TUR, or combined regarding to the control (3.7 ± 0.20, 41.33 ± 0.05, 3.36 ± 0.09, and 2.23 ± 18.56, respectively.

### 3.8. Outcomes of Challenges with A. hydrophila 

**Figure 4:**
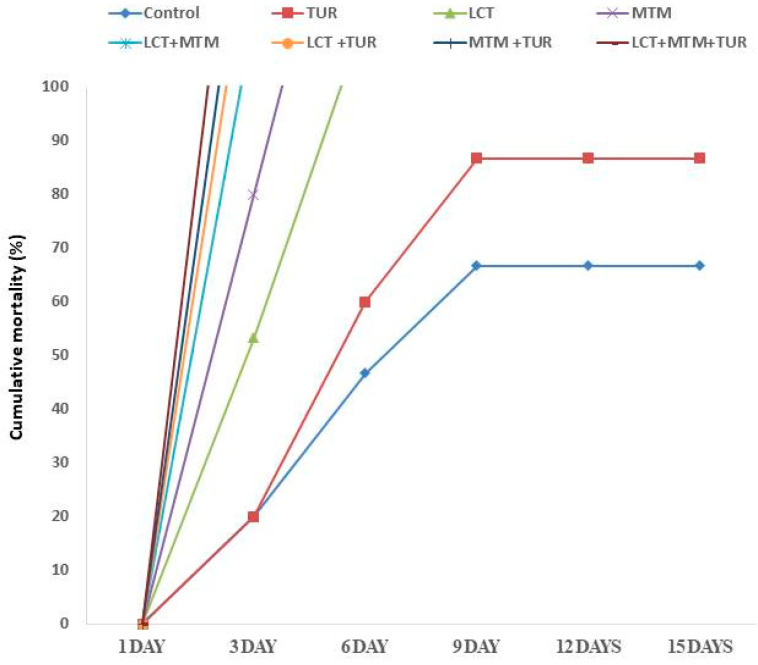
The cumulative mortality (%) of *O. niloticus* after exposure to LCT and/or MTM (1/20 of LC50), fed a diet containing 1% taurine (TUR) for 60 days. Each line represents a group’s cumulative mortality (%) from days 1 to 15 post-challenge by *A. hydrophila* infection.

Appetite failure, lethargy, erratic behavior, and hemorrhages on all body surfaces were observed in the control group’s challenged fish and in the LCT and/or MTM classes. Post-challenge, the mortality was recorded in all groups (Figure 4), and the highest mortality percent was recorded in LCT + MTM-exposed fish (86.67%) followed by LCT (80%), MTM (73.33%), and control groups (66.67%). On the other hand, the fish fed on a TUR-supplemented diet showed reduced mortality percentages of 46.67%, 40%, and 53.33% in LCT + TUR, MTM + TUR, and LCT+ MTM + TUR groups, respectively. The lowest mortality was recorded in TUR-fed fish (66.67%) relative to the control fish.

## 4. Discussion

Aquatic organisms, especially fish, are exposed to major hazards of disruption in their immune responses because of their constant exposure to various toxic chemicals, such as pesticides, that are released into the aquatic environment [2,49]. Hence, the impacts of LCT and/or MTM exposure for 60 days on growth and immunological parameters and the alterations in the expression of immune-related genes were investigated in *O. niloticus* fish, elucidating an immune depressive effect. Additionally, the mitigating role of a 1%-TUR-supplemented diet, co-administered to intoxicated fish to alleviate the toxic effects of these insecticides, was investigated.

In this study, low growth performance was observed in *O. niloticus* exposed to LCT and/or MTM, and there was an apparent reduction for LCT + MTM-exposed fish. These findings could be attributed to the metabolic changes in protein and carbohydrate metabolism of fish induced by toxic stress, which led to the consumption of energy storage to detoxify the toxicant or repair mechanisms; hence, little energy was available for growth. A reduction in growth has previously been reported for LCT exposed in *O. niloticus* [30]; likewise for cypermethrin (a member of pyrethroids family) given to common carp [50], and MTM given to *Hoplobatrachus rugulosus* [25]. 

Remarkably, concurrent TUR administration with LCT and/or MTM in the diet improved growth. The growth-promoting effect of TUR could be because TUR enhances amino acid uptake, energy utilization, protein, and lipid synthesis [51]. Moreover, TUR could enhance feed palatability and intake and digestive enzyme secretion [43]. Consequently, TUR supplementation optimizes energy utilization, accelerates growth, and improves feed utilization and digestibility, as reported by Al-Feky, El-Sayed, and Ezzat [38]. Besides, TUR makes the feed highly palatable because of its animal origin, whereas most formulas used in fish meals are of plant origin, which makes them less palatable for fish, thereby decreasing food consumption [34]. Similar growth-enhancing effects of TUR-supplemented diet were reported for white shrimp, *Litopenaeus vannamei* [52]; *O. niloticus* [38]; orange-spotted grouper, *Epinephelus coioides* [53]; and African catfish, *Clarias gariepinus* [54,55]. Moreover, a TUR-supplemented diet could improve the growth of common carp exposed to salinity stress (10 ppt range) for eight weeks [54].

Our results showed increases in mortality rates for LCT and/or MTM-exposed fish, with the highest rate being observed in the LCT + MTM-exposed group. Moreover, imbalances in swimming behavior and respiratory distress manifested by the rapid opercular movement were induced upon LCT and/or MTM exposure. These alterations could be attributed to respiratory impairment and osmoregulatory failure, as the first route of entry for contaminants in fish is through the gills [1]. Moreover, the erratic swimming of fish might have been due to the impact of these pesticides on the central nervous system. This behavior was confirmed in this study by inhibition of AchE activity, thereby hindering synchronization between nervous and muscular junctions, leading to diminished fish performance and swimming capabilities [56,57]. Similar impairments in behavior and locomotion were reported for LCT exposure in freshwater catfish, *Clarias batrachus* [58], and *O. niloticus* [5,30]. Inhibition of AChE activity was also reported for exposure of *O. niloticus* to LCT for 15 days, and in muddy loach, *Misgurnus anguillicaudatus*, for MTM exposure for 21 days [59], suggesting a neurotoxic effect. On the contrary, a TUR-supplemented diet in LCT and/or MTM-exposed fish reduced the mortality rate and improved clinical signs, owing to its potent antioxidant activity that could protect cell organelles from oxidative stress induced by pesticides [60]. In common carp exposed to salinity stress, the lowest mortality was recorded in fish fed on a TUR-supplemented diet for 8 weeks [55].

Hematological indices are essential and sensitive indicators for fish health; they screen responses to stress induced by chemical pollutants [61]. In the current study, LCT and/or MTM exposure exerted marked reductions in hematological parameters (RBC count, PCV percentage, Hb, and MCV). The worst effect was evident in the combination group. This effect could be explained by the erythropoiesis suppression and increased erythrocyte destruction rate induced by oxidative stress—which enhances the peroxidation of unsaturated fatty acids of a RBC’s membrane. Declines in Hb and PCV values as a compensatory response to maintain gas exchange in destructed gills to reduce oxygen-carrying capacity were noticed [62]. Furthermore, LCT and/or MTM exposure significantly (*p* < 0.001) decreased the total WBC counts, especially of lymphocytes, elucidating a stress condition and suppressing a non-specific immune response in *O. niloticus*. These reductions could be attributed to the cytotoxic effects of LCT and MTM in bone marrow cells and RBCs. They could result from excess reactive oxygen species (ROS) generation that hinders the antioxidant defense system and oxidizes membrane phospholipids of RBCs and WBCs, resulting in loss of membrane integrity. Therefore, the occurrence of cellular apoptosis [63] was proven by the increased level of MDA and the reduction of antioxidant enzymes in our study. Alterations in hematological variables were recorded in *O. niloticus* exposed to MTM and LCT [30,64]. TUR supplementation in LCT and/or MTM-exposed fish modulated these indices; such effects might be related to the key roles played by TUR in the synthesis of hemoglobin and stabilization of RBC homeostasis shown in some studies [65,66] that reported improved hematological status of Totoaba juveniles, *Totoaba macdonaldi*, fed a TUR diet. Similar improvements in the hematological indices were recorded in *O. niloticus* exposed to LCT and fed on a 2%-thyme-supplemented diet for 30 days. Additionally, the RBCs of *O. niloticus* fish exposed to MTM and treated with brown seaweed (*Sargassum polycystum*) extract showed a statistically significant (*p* < 0.001) elevation in number compared with the untreated group [64].

The inherent immune system of a fish, the first line of pathogen defense, is impaired upon exposure to different water toxicants [67]. The bacterial cell wall lysozyme, a major member of this mechanism, stimulates the complementary system. Nitric oxide (NO) is a known part of a macrophage-forming innate immune system. Complement-3 (C3), a key component, also displays various immune functions, such as the mediation of the inflammatory response and the destruction of invading pathogens. Moreover, the key element of the humoral immune response is antibodies, and immunoglobulin M (IgM) is the key antibody in fish [68,69]. There was a marked decline in the immunological response (lysozyme activity, NO, C3, and IgM) in fish exposed to LCT and/or MTM. Our results support previous findings that LCT exposure induced a substantial decrease in the immune response of *O. niloticus* after 30 days [30]. Feeding a TUR-supplemented diet to LCT and/or MTM-exposed fish considerably restored their immune parameters, suggesting enhanced immunological defense and confirming the immunostimulatory effect of TUR. Previous studies have documented improved immune responses for TUR-supplemented young grass carp [70], European sea bass [71], and *O. niloticus* [54].

The serum proteins are fundamental indicators of fish health and immune status. Their levels are modulated under different physiological and pathological conditions. This study showed that LCT and/or MTM exposure decreased total protein, albumin, and globulin levels, especially α-globulin. This could be explained by impaired liver function because of liver cell destruction by pesticides [72]. Similarly, one study showed lowered blood protein values of African catfish from LCT exposure [73]. Supplementation of the diet with TUR significantly (*p* < 0.001) improved these parameters in LCT and/or MTM exposed fish, indicating the ability of TUR to restore damaged hepatocytes and repair liver function regarding protein synthesis, and hence, TUR elevated fish immunity as previously reported [74]. Another study [75] highlighted significant increases (*p* < 0.001) in the protein profiles of black sea bream and *O. niloticus* fed TUR enriched diets.

Oxidative stress results from a wide variety of environmental xenobiotics, such as pesticides, that can produce ROS, leading to lipid peroxidation, modulation of gene expression, and protein oxidations. MDA is the major secondary lipid peroxidation output of polyunsaturated fatty acids [63]. Additionally, the presence of 8-OHdG is considered a biomarker of DNA damage induced by oxidative stress [76]. The most remarkable enzymes for the detoxification of ROS are CAT, SOD, and GPX; hence, assessing these biomarkers is crucial in aquatic organisms [77,78]. In this study, LCT and/or MTM- exposure induced oxidative damage by lowering antioxidant enzyme activity and enhancing lipid peroxidation (MDA) and DNA damage (8-OHdG) in *O. niloticus*. The combination group showed a marked effect. Similarly to our findings, LCT has been found to induce oxidative stress in different fish species, such as in *Prochilodus lineatus* [18], zebrafish [79], and *O. niloticus* [30]. MTM can produce free radicals, which can have a marked impact on the antioxidant defense system in *O. niloticus* [80]. Additionally, one study [25] reported increased MDA levels in the liver and kidney of *H. rugulosus* exposed to MTM. Meanwhile, TUR administration alone or in combination with LCT and/or MTM exposure in *O. niloticus* could enhance the antioxidative ability to combat a high degree of oxidative harm. TUR has been shown to be a potent antioxidant, as a radical scavenger free of oxygen, which reduces lipid peroxidation and protects tissues against oxidative harm [33]. Several studies have reported that a TUR-enriched diet could improve antioxidant activity and decrease MDA levels in yellow catfish, common carp, and African catfish, when exposed to different stress [54,55,81].

An evaluation of immunotoxicity in the spleen of *O. niloticus* was achieved by the immunotoxicity impact of LCT/ MTM (as a main immune organ). Exposure to LCT and/or MTM upregulated TNF-α, IL-1β, CC, and CXC genes and down-regulated IL-10 expression, as was clarified by the mRNA transcript levels, suggesting the impacts of these pesticides at the molecular level. The recorded impairment suggested the immunosuppressive effect of these pesticides, consequently ever-increasing the susceptibility of fish to pathogens and causing a higher mortality rate in those fish. The expression of cytokines (IL-1β and TNF-α) as a vital part of the immune system is modulated by infection or inflammation, so assessing their expression levels may help explain the mechanisms of specific contaminants’ toxicity [82]. Environmental toxins have been found to be a disruptive cause of the expression patterns of those genes linked to the immune response. TUR supplementation helped inhibit the immune-related encoding gene in fish exposed to LCT and/or MTM, indicating an enhancement of the immune response. Two studies [30,83] reported modulated immune-related genes of *O. niloticus* exposed to LCT for 30 days. Additionally, brown seaweed was previously reported to mitigate the genotoxic effect of MTM in *O. niloticus* [64]. CC and CXC chemokines are the key immune regulators and act as a critical bridge between innate immunity and adaptive immunity. In addition to modulation of an immune reaction, CC and CX specifically induce leukocyte recruitment and differentiation in recruited cells [84]. Therefore, the up-regulation of their expression in the immune tissues (kidney and spleen) of fish plays an important role in the immune response and is associated with the exposure of fish to different environmental pollutants and infections [85].

The host resistance challenge test is the most critical assay to confirm and assess the organism’s immune functions [86]. Here, *O. niloticus* was further challenged with *A. hydrophila* after exposure to LCT and/or MTM for 60 days. The results showed that the mortality rate was elevated in the LCT and MTM groups. In contrast, TUR supplementation alone significantly reduced the mortality rate. These findings have shown that the immuno-modulatory activity of TUR upgraded the disease resistance of *O. niloticus*. This may be related to the augmented non-specific immune indices (lysozyme, NO, IgM, and C3) and the up-regulation of immune-related genes. Similar outcomes were recorded in silver catfish exposed to glyphosate (GLP) or atrazine (ATZ) and challenged with *A. hydrophila* [87], and common carp exposed to ATZ and challenged with *Aeromonas sobria* [88].

## 5. Conclusions

In the present work, *O. niloticus* exposed to LCT and/or MTM exhibited changes in several immunological parameters, including leukocyte count; complement 3 concentration; antioxidant enzyme concentrations; and mRNA expression for TNF-α, IL1B, CC, and CXC. The resistance of fish against the experimental challenge with *A. hydrophila* was reduced due to exposure to LCT and/or MTM. The combination of both insecticides (LCT + MTM) showed maximum toxicity in *O. niloticus* after 60 days of exposure. This study also clarified that the toxic effect of LCT is more severe than exposure to MTM in *O. niloticus*. Upon supplementing the diet with 1% TUR, antioxidant defense and innate immunity components were promoted. Consequently, the immune status of *O. niloticus* was boosted to counteract the immunosuppressive impact of LCT/MTM; additionally, it enhanced the growth and bodily condition of fish. Taurine is hence a promising dietary supplement for *O. niloticus* in aquaculture.

## Figures and Tables

**Table 1:** Proximate chemical composition of the basal diet (%).

Ingredients	%
Yellow corn	30
Soybean meal, 48%	20
Meat meal high fat, 50%	18
Wheat flour	10
Fish meal, 60%	15
Vegetable oil	5.5
Vitamins and minerals mixture ^1^	1.5
Total	100
Chemical analysis (%) ^2^	
DM	86.02
CP	32.02
EE	9.93
CF	1.74
Ash	10.13
NFE	35.97
DE, Kcal/ kg diet ^3^	2867.48

^1^ Vitamin and Mineral mixture: Each 1 kg contains: Vit. D3 8600 I.U, vit. A 580000 I.U, vit C 0.1 mg, vit. E. 720 mg, vit B1 58 mg, vit. K3 142 mg, vit B2 34 mg, vit. B12 58 mg, vit. B6 34 mg, Pantothenic acid 8 mg, Folic acid 86 mg, Zinc methionine 3000 mg, Manganese sulfate 65 mg, Copper sulfate 3400 mg, Iron sulfate 2000 mg, Sodium selenite 25 mg, Cobalt sulfate 572 mg, Calcium iodide 25 mg, Calcium carbonate (Carrier substance) till one kg. ^2^ According to NRC (2011). ^3^ Digestible energy.

**Table 2:** Acute 96-h toxicity of lambda-cyhalothrin (LCT) and methomyl (MTM) in O*. niloticus.*

		LCT			
Intercept ± S.E.	Slope ± S.E.	95 % Confidence Limit		Conc (µg/L)	Point
		upper	lower		
−3.446 ± 0.455	2.167 ± 0.281	0.140	0.748	0.517	LC 1
		0.555	1.005	0.831	LC 5
		0.774	1.145	0.999	LC 10
		0.920	1.241	1.112	LC 15
		1.491	1.692	1.590	LC 50
		1.935	2.272	2.069	LC 85
		2.030	2.418	2.182	LC 90
		2.169	2.637	2.349	LC 95
		2.426	3.053	2.664	LC 99
**Methomyl (MTM)**					
−2.401 ± 0.262	0.006 ± 0.001	83.386	−93.678	12.670	LC 1
		181.548	51.595	128.463	LC 5
		235.159	127.758	190.192	LC 10
		272.273	178.202	231.840	LC 15
		448.241	372.456	407.941	LC 50
		658.665	532.254	584.042	LC 85
		710.568	567.909	625.690	LC 90
		788.096	620.155	687.419	LC 95
		934.650	717.036	803.212	LC 99

**Table 3:** Primer sequences used in qRT-PCR.

Gene	Forward Primer (5′–3′)	Reverse Primer (5′–3′)	Accession No
*TNF-α*	CCAGAAGCACTAAAGGCGAAGA	CCTTGGCTTTGCTGCTGATC	NC_031985.2
*IL-1b*	TGGTGACTCTCCTGGTCTGA	GCACAACTTTATCGGCTTCCA	DQ061114.1
*IL-10*	CTGCTAGATCAGTCCGTCGAA	GCAGAACCGTGTCCAGGTAA	NC031970.1
*CC-chemokine*	ACAGAGCCGATCTTGGGTTACTTG	TGAAGGAGAGGCGGTGGATGTTAT	FF279635.1
*CXC-chemokine*	CTATCCATGGAGCCTCAGGT	CACTCCAGAGATCAAAGCAGTTCC	XM_003452201
*GAPDH*	CCGATGTGTCAGTGGTGGAT	CTTCTTGAGCGTGGCAATAA	NC_031976.2

TNF-α—tumor necrosis factor alpha, IL-1β—interleukin-1 beta, IL-10—interleukin-10.

**Table 4:** Comparative and combined effects of LCT and MTM (1/20 of LC_50_) on growth performance hematological indices on *O. niloticus* and the role of Taurine (1% in diet). For 60 days (*n* = 60).

Parameter	Control	TUR	LCT	MTM	LCT + MTM	LCT + TUR	MTM + TUR	LCT + MTM + TUR
Initial body weight (g)	30.24 ± 0.005	30.28 ± 0.0 ^ns^	30.28 ± 0.1^ns^	30.23 ± 0.18 ^ns^	30.48 ± 0.0 ^ns^	30.25 ± 0.22 ^ns^	30.49 ± 0.15 ^ns^	30.60 ± 0.05 ^ns^
Final body weight (g)	57.12 ± 0.58	58.60 ± 0.91 ^ns^	42.54 ± 0.64 **	44.96 ± 1.10 **	37.04 ± 0.49 ***	48.34 ± 0.78 ^##^	40.84 ± 0.38 ^##^	44.22 ± 0.24 ^##^
Weight gain (g)	26.88 ± 0.56	28.32 ± 0.91 *	12.26 ± 0.65 ***	14.66 ± 1.24 **	6.70 ± 0.52 ***	18.06 ± 0.70 ^###^	10.38 ± 0.47 ^###^	13.62 ± 0.30 ^###^
SGR (%)	1.13 ± 0.02	1.18 ± 0.03 ^ns^	0.61 ± 0.03 **	0.70 ± 0.05 **	0.36 ± 0.03 ***	0.83 ± 0.03 ^##^	0.52 ± 0.02 ^##^	0.66 ± 0.01 ^###^
K (condition factor)	0.46 ± 0.02	0.43 ± 0.01 ^ns^	0.35 ± 0.02 *	0.37 ± 0.01 *	0.41 ± 0.02 *	0.43 ± 0.02 ^#^	0.35 ± 0.01 ^ns^	0.36 ± 0.01 ^ns^
No of mortality	0/60	0/60	31/60	26/60	37/60	18/60	12/60	21/60
Mortality %	0	0	51.66	43.33	61.67	30	20	35
**Hematological Indices**								
RBCs (106/mm^3^)	3.03 ± 0.08	3.27 ± 0.06 ^ns^	1.38 ± 0.25 ***	1.20 ± 0.04 ***	0.70 ± 0.04 ***	2.53 ± 0.06 ^##^	2.10 ± 0.04 ^##^	1.33 ± 0.13 ^##^
Hb (gm/dL)	9.43 ± 0.22	9.10 ± 0.04 ^ns^	3.50 ± 0.23 ***	4.93 ± 0.24 ***	2.21 ± 0.04 ***	6.57 ± 0.08 ^###^	5.98 ± 0.27 ^##^	4.30 ± 0.12 ^##^
PCV (%)	27.00 ± 0.71	28.33 ± 0.85 ^ns^	14.33 ± 0.62 ***	19.00 ± 1.08 **	8.00 ± 0.41 ***	21.00 ± 0.41 ^##^	18.68 ± 0.24 ^ns^	14.00 ± 0.41 ^##^
MCV(fl)	104.6 ± 1.60	101.5 ± 1.14 ^ns^	170.8 ± 2.00 ***	161.7 ± 1.70 ***	127.9 ± 2.32 ***	143.3 ± 1.05 ^###^	136.6 ± 1.52 ^##^	118.8 ± 0.23 ^#^
WBCs (10^3^/mm^3^)	6.13 ± 0.08	5.47 ± 0.17 ^ns^	2.50 ± 0.16 ***	3.87 ± 0.12 **	1.78 ± 0.05 ***	4.67 ± 0.15 ^###^	4.70 ± 0.04 ^##^	3.47 ± 0.05^#^
Lymphocytes (10^3^/mm^3^)	2.8 ± 0.04	3.0 ± 0.13 ^ns^	1.1 ± 0.08 ***	2.0 ± 0.06 **	0.9 ± 0.02 ***	1.8 ± 0.04 ^##^	2.2 ± 0.04 ^#^	1.4 ± 0.04 ^##^
Heterophils (10^3^/mm^3^)	1.82 ± 0.01	1.68 ± 0.07 ^ns^	1.05 ± 0.24 **	1.30 ± 0.04 *	0.70 ± 0.04 ***	1.70 ± 0.04 ^###^	1.43 ± 0.02 ^#^	1.37 ± 0.02 ^##^
Eosinophils (10^3^/mm^3^)	0.35 ± 0.03	0.33 ± 0.01 ^ns^	0.14 ± 0.03 **	0.20 ± 0.04 *	0.13 ± 0.03 **	0.23 ± 0.02 ^#^	0.20 ± 0.04 ^#^	0.27 ± 0.02 ^#^
Monocytes (10^3^/mm^3^)	0.62 ± 0.01	0.62 ± 0.01 ^ns^	0.41 ± 0.03 **	0.51 ± 0.01 *	0.19 ± 0.04 **	0.37 ± 0.02 ^ns^	0.20 ± 0.04 ^ns^	0.10 ± 0.00 ^ns^

The values are shown in means ± SEs. One-way ANOVA followed by Tukey’s post hoc tests to measure the significance of differences between groups. * or ^#^—*p* ≤ 0.05 (low significance); ** or ^##^— *p* ≤ 0.01 (moderate significance); *** or ^###^—*p* < 0.001 (highly significant difference). ns: *p* > 0.05 indicates a non-significant difference. The significance was applied as follows: LCT, MTM, and LCT + MTM versus control and represented by the (*) sign; LCT versus the LCT + TUR group, MTM versus (MTM + TUR), and LCT + MTM versus LCT + MTM +TUR group, represented by (#) sign. * is the same value as #. The difference is only in shape.

**Table 5:** Comparative and combined effects of LCT and MTM (1/20 of LC50) on AchE activity, OHDG, immunological indices, serum protein profile, mortality number, and percent post-infection for *O. niloticus*, and the role of taurine (1% in diet). Sixty days (*n* = 60).

Parameter	Control	TUR	LCT	MTM	LCT + MTM	LCT + TUR	MTM + TUR	LCT + MTM + TUR
AchE	9.43 ± 0.22	9.10 ± 0.04 ^ns^	3.50 ± 0.23 ***	4.93 ± 0.24 ***	2.25 ± 0.06 ***	6.57 ± 0.08 ^###^	5.98 ± 0.27 ^##^	4.30 ± 0.12 ^###^
8OHDG	26.67 ± 1.5	30.67 ± 1.03 ^ns^	85.33 ± 0.62 ***	76.67 ± 0.62 ***	106.5 ± 0.65 ***	44.0 ± 0.41 ^##^	56.0 ± 0.41 ^##^	64.0 ± 1.08 ^###^
Immunoglobulin M (mg/dL)	121.3 ± 0.62	125.7 ± 2.46 ^ns^	58.33 ± 1.02 ***	64.07 ± 4.28 ***	41.13 ± 0.29 ***	65.93 ± 1.94 ^##^	76.73 ± 0.56 ^##^	53.43 ± 1.02 ^###^
Lyzozyme activity	30.60 ± 0.37	29.57 ± 0.37 ^ns^	13.67 ± 0.21 ***	17.10 ± 0.53 ***	10.94 ± 0.20 ***	19.60 ± 0.51 ^###^	21.40 ± 0.39 ^###^	15.61 ± 0.24 ^###^
Complement 3 (ug/mL)	78.6 ± 0.45	75.9 ± 0.72 ^ns^	48.8 ± 1.13 ***	56.53 ± 0.34 ***	36.26 ± 0.73 ***	61.67 ± 0.58 ^###^	66.9 ± 0.19 ^###^	45.23 ± 0.41 ^###^
Nitric oxide (μmol/L)	66.47 ± 0.31	67.50 ± 0.60 ^ns^	38.77 ± 0.51 **	43.03 ± 0.89 **	29.69 ± 0.15 ***	56.43 ± 0.27 ^##^	54.27 ± 1.38 ^###^	42.63 ± 0.82 ^#^
Total protein (g/dL)	5.40 ± 0.08	5.40 ± 0.16 ^ns^	4.60 ± 0.08 **	4.80 ± 0.08 **	5.00 ± 0.16 **	4.67 ± 0.17 ^ns^	5.13 ± 0.21 ^#^	4.87 ± 0.12 ^#^
Albumin (A)(g/dL)	2.63 ± 0.06	2.53 ± 0.05 ^ns^	2.27 ± 0.08 *	2.47 ± 0.05 ^ns^	2.61 ± 0.15 ^ns^	2.23 ± 0.08 ^ns^	2.57 ± 0.17 ^ns^	2.40 ± 0.08 ^ns^
Globulin (G)(g/dL)	2.77 ± 0.02	2.87 ± 0.12 ^ns^	2.27 ± 0.02 *	2.23 ± 0.05 *	2.11 ± 0.04 *	2.30 ± 0.04 ^#^	2.43 ± 0.05 ^#^	2.33 ± 0.08 ^#^
A/G	0.95 ± 0.02	0.89 ± 0.03 ^ns^	1.00 ± 0.03 ^ns^	1.11 ± 0.02 *	1.24 ± 0.08 **	0.97 ± 0.03 ^ns^	1.05 ± 0.05 ^ns^	1.04 ± 0.06 ^#^
α-globulin−1(g/dL)	0.79 ± 0.01	0.77 ± 0.01 ^ns^	0.30 ± 0.04 **	0.40 ± 0.04 **	0.14 ± 0.02 ***	0.44 ± 0.02 ^#^	0.40 ± 0.04 ^ns^	0.25 ± 0.02 ^##^

The values are shown as means ± SEs. One-way ANOVA followed by Tukey’s post hoc tests to measure the significance of differences between groups. * or ^#^—*p* ≤ 0.05 (low significance); ** or ^##^— *p* ≤ 0.01 (moderate significance); *** or ^###^—*p* < 0.001 (highly significant difference). ns: *p* > 0.05 indicates a non-significant difference. The significance was applied as follows: LCT, MTM, and LCT + MTM versus control and represented by the (*) sign; LCT versus the LCT + TUR group, MTM versus (MTM + TUR), and LCT + MTM versus LCT + MTM +TUR group, represented by (#) sign. * is the same value as #. The difference is only in shape.

## Data Availability

Not applicable.
